# Exclusive Temporal Stimulation of IL-10 Expression in LPS-Stimulated Mouse Macrophages by cAMP Inducers and Type I Interferons

**DOI:** 10.3389/fimmu.2019.01788

**Published:** 2019-08-06

**Authors:** Orna Ernst, Yifat Glucksam-Galnoy, Bibek Bhatta, Muhammad Athamna, Iris Ben-Dror, Yair Glick, Doron Gerber, Tsaffrir Zor

**Affiliations:** ^1^Department of Biochemistry & Molecular Biology, School of Neurobiology, Biochemistry & Biophysics, Tel Aviv University, Tel Aviv, Israel; ^2^Triangle Regional Research and Development Center, Kafr Qara, Israel; ^3^The Nanotechnology Institute, Bar-Ilan University, Ramat Gan, Israel

**Keywords:** IL-10 promoter, cAMP, type I interferons, IL-10 expression, lipopolysaccharide, cAMP response element, CREB, toll-like receptor 4

## Abstract

Expression of the key anti-inflammatory cytokine IL-10 in lipopolysaccharide (LPS)-stimulated macrophages is mediated by a delayed autocrine/paracrine loop of type I interferons (IFN) to ensure timely attenuation of inflammation. We have previously shown that cAMP synergizes with early IL-10 expression by LPS, but is unable to amplify the late type I IFN-dependent activity. We now examined the mechanism of this synergistic transcription in mouse macrophages at the promoter level, and explored the crosstalk between type I IFN signaling and cAMP, using the β-adrenergic receptor agonist, isoproterenol, as a cAMP inducer. We show that silencing of the type I IFN receptor enables isoproterenol to synergize with LPS also at the late phase, implying that autocrine type I IFN activity hinders synergistic augmentation of LPS-stimulated IL-10 expression by cAMP at the late phase. Furthermore, IL-10 expression in LPS-stimulated macrophages is exclusively stimulated by either IFNα or isoproterenol. We identified a set of two proximate and inter-dependent cAMP response element (CRE) sites that cooperatively regulate early IL-10 transcription in response to isoproterenol-stimulated CREB and that further synergize with a constitutive Sp1 site. At the late phase, up-regulation of Sp1 activity by LPS-stimulated type I IFN is correlated with loss of function of the CRE sites, suggesting a mechanism for the loss of synergism when LPS-stimulated macrophages switch to type I IFN-dependent IL-10 expression. This report delineates the molecular mechanism of cAMP-accelerated IL-10 transcription in LPS-stimulated murine macrophages that can limit inflammation at its onset.

## Introduction

The TLR4 ligand, LPS, stimulates macrophages to produce and secrete multiple pro-inflammatory mediators (1). Expression of the anti-inflammatory cytokine IL-10 peaks with a delay that is due to the essential involvement of LPS-stimulated type I interferons (IFN) that act in an autocrine and paracrine manner (2–7). For example, in LPS-stimulated RAW264.7 macrophages, there is an approximately 10 h time gap between the TNFα and IL-10 expression peaks (8). Yet, anti-inflammatory macrophages, characterized by enhanced IL-10 expression, can be also generated by a combination of LPS and a second signal, such as an IgG immune complex, apoptotic cell remnants, or a cAMP inducer (1). We have previously shown that short co-stimulation of macrophages with LPS and a cAMP inducer results in synergistic IL-10 transcription, while either stimulus alone is largely ineffective (9). Synergistic IL-10 expression has also been demonstrated in macrophages stimulated by a cAMP inducer and agonists of other TLRs (9, 10). Recently, we further demonstrated that the enhancement of LPS-stimulated IL-10 expression by cAMP and by autocrine type I IFN is temporally distinct (11). Exogenous agents that elevate cAMP, such as the β-adrenergic receptor (β-AR) agonist isoproterenol or the phosphodiesterase (PDE)-4 inhibitor rolipram, synergize with early type I IFN-independent IL-10 expression by LPS, but in contrast, are unable to amplify the late type I IFN-dependent activity (11). In the current study we explored the mechanism of IL-10 expression temporal regulation at the promoter level.

LPS-stimulated IL-10 induction strictly depends on the p38 pathway, which inhibits IL-10 mRNA decay (12, 13). Additionally, p38 activates several transcription factors (TFs), among them Sp1 which has been shown to be involved in IL-10 expression (14). It has also been suggested that LPS-stimulated p38 activates CREB by MSK1/2-mediated phosphorylation on S133 (15), an event considered to be requisite for CREB function (16). However, we have shown that cAMP-stimulated PKA phosphorylates and activates CREB, whereas LPS-stimulated p38-MSK1/2 phosphorylates CREB but fails to activate CRE-dependent transcription (17), indicating that phosphorylation of CREB is required but not sufficient for its transcriptional activity (18). Indeed, the CREB-regulated transcription coactivator 3 (CRTC3) translocates to the nucleus following cAMP-dependent PKA activation, but not in response to LPS, where it cooperates with CREB in amplification of LPS-induced IL-10 expression (19). A cross-talk between LPS and cAMP signaling might occur also at the level of p38 activation, as cAMP induction in LPS-stimulated BMDM increased expression of the MAPK phosphatase DUSP1, leading to reduced MAPK activity (20). As expected, IL-10 expression is elevated in DUSP1-deficient macrophages in a p38-dependent manner (21). As the cAMP-DUSP1 axis down-regulates p38 activity and IL-10 expression in LPS-stimulated macrophages (20), whereas overall cAMP strongly amplifies IL-10 expression (11), we hypothesized that cAMP magnifies LPS-induced IL-10 via a p38-independent mechanism.

The repertoire of signaling pathways which are employed to induce IL-10 depends on the studied species and cell type (22–24). The TFs shown to be involved in LPS-stimulated IL-10 induction in murine macrophages, and whose respective response elements were mapped on the mouse IL-10 promoter, are: C/EBP (25), Sp1 and Sp3 (26, 27), STAT1 and STAT3 (3), KLF4 (28), and NFκB p50 (29). Brightbill et al. (26) used a series of 5′-deletion mutants and point mutations of the mouse IL-10 promoter reporter to show that the Sp1 site, located at −89/−78 bp relative to the transcription start site (TSS), is primarily responsible for IL-10 reporter transcription in RAW264.7 macrophages stimulated by LPS (alone) for a long period of 24 h (26). The synergistic IL-10 transcription displayed by LPS and cAMP inducers (9, 11), together with the suppressive effect of CREB deficiency on IL-10 expression in mouse macrophages (19, 30), led us to hypothesize that LPS-stimulated Sp1 cooperates with cAMP-stimulated CREB at the mouse IL-10 promoter. While the location of CRE at the mouse IL-10 promoter remains elusive, Platzer et al. (31) stimulated human THP-1 monocytes for 24 h with cell-permeable cAMP (alone) and identified two functional and two non-functional CREs in the human IL-10 promoter. However, only one of these CRE sites is conserved in the mouse promoter, and importantly—their relevance to IL-10 expression in LPS-stimulated cells has not been explored. Binding of phosphorylated CREB to the proximal region of the mouse IL-10 promoter has been demonstrated, but the precise location of CRE has not been revealed (32, 33).

The above reports examined transcriptional regulation of the IL-10 promoter in cells stimulated for a prolonged period with either LPS or a cAMP inducer alone. The objective of the present study was to identify the mouse IL-10 promoter elements that mediate synergistic induction by cAMP at the early phase in co-stimulated macrophages, and to asses why up-regulation by cAMP is lost upon switch of LPS-stimulated macrophages to type I IFN-dependent IL-10 expression. We found that type I IFN receptor silencing enabled synergism between LPS and cAMP also at the late phase, suggesting that type I IFN stimulate IL-10 expression at the late phase via a mechanism which is not amenable for up-regulation by the cAMP pathway. We then identified a novel set of two functionally-dependent CREs at the mouse IL-10 promoter that is activated by the cAMP pathway and drives IL-10 reporter transcription in a cooperative manner with the Sp1 site, which is mainly constitutive at the early phase and then further activated by LPS via type I IFN at a later stage. Our results suggest that accelerated IL-10 transcription achieved by synergism between cAMP inducers and type I IFN-independent LPS signaling can limit inflammation at its onset in specific contexts.

## Materials and Methods

### Reagents and Plasmids

Lipopolysaccharide (LPS; *Escherichia coli* serotype 055:B5) and isoproterenol were purchased from Sigma-Aldrich (St. Louis, MO). L-glutamine and penicillin-streptomycin-nystatin were purchased from Biological Industries (Beit Haemek, Israel). DMEM, OptiMEM and FBS were purchased from Gibco. BSA was purchased from Amresco (Solon, OH). The ELISA reagents set for IL-10 was purchased from R&D Systems (Minneapolis, MN). The rabbit anti-mouse CREB and monoclonal anti-mouse tubulin antibodies were from Cell Signaling Technology (Danvers, MA) and Santa Cruz Biotechnology (Santa Cruz, CA), respectively. Infrared dye-labeled secondary antibodies and blocking buffer were from Li-Cor Biosciences (Lincoln, NE). Immobilon-FL polyvinylidene fluoride (PVDF) membranes were from Millipore (Billerica, MA). The full-length (−1,538/+64) mouse IL-10 promoter luciferase reporter gene construct and the set of 5′-deletion mutants were a kind gift from Dr. S. Smale (26) and the dominant negative construct named A-CREB was generously given by Dr. C. Vinson (34). All vectors were amplified using DH10B bacteria (Invitrogen, Carlsbad, CA), and purified using Endofree Plasmid Maxi Kit (Qiagen, Hamburg, Germany). HD-fugene, Lipofectamine2000 and TransIT2020 transfection reagents were purchased from Roche (Mannheim, Germany), Invitrogen (Carlsbad, CA) and Mirus Bio (Madison, WI), respectively. Dual-luciferase reporter assay kit was from Promega (Fitchburg, WI). The siRNA against CREB (5′-GCAAGAGAAUGUCGUAGAA-3′) and a scrambled control sequence were purchased from Bioneer (Daejeon, Korea). Mouse IFNα was from Miltenyi Biotec (Bergisch Gladbach, Germany). PCERA-1 was kindly supplied by Dr. Nathanael Gray.

### Cell Culture

Mouse RAW264.7 macrophage cells were obtained from American Type Culture Collection (ATCC, Rockville, MD). A RAW264.7 cell line stably expressing shRNA against CREB1a was generously given by Dr. I.D.C. Fraser (30). The cells were grown to 80–90% confluence in DMEM medium supplemented with 8 mM L-glutamine, 100 U/ml penicillin, 100 μg/ml streptomycin and 1,250 U/ml nystatin (hereafter culture medium), and with 10% FBS, at 37°C in a humidified incubator with 5% CO_2_.

### IL-10 Release Assay

RAW264.7 macrophages were maintained for 48 h prior to the experiment in 96-well plates, at 1.0·10^5^ cells per well, in culture medium supplemented with 5% FBS, up to a confluence of 90%. The culture medium was replaced 2 h before treatment in order to avoid the artifact of medium replacement on signaling (35). The cells were stimulated with LPS (10 ng/ml) and/or isoproterenol (1 μM) at 37°C for 3–24 h. IL-10 secretion to the medium were measured with commercially available ELISA reagents sets, according to the manufacturer's instructions, using a microplate reader (Bio-Tek, Winooski, Vermont). The samples were stored at −80°C until used.

### Transfection and Reporter Gene Assay

RAW264.7 macrophages were grown for 24 h in 12-well plates, at 3·10^5^ cells per well, in culture medium supplemented with 10% FBS. The cells were then transfected for 24 h with 0.6 μg of reporter plasmid and 0.2 μg of Herpes Simplex Virus TK-renilla luciferase (for normalization), and where indicated—also with a dominant negative (A-CREB), silencing (sh-IFNαR1) or control construct. The plasmids were initially incubated with HD-fugene or TransIT2020 transfection reagent in OptiMEM for 15 min at room temperature. Following transfection, the cells were washed and stimulated with LPS (10 ng/ml) and/or isoproterenol at 37°C for 3–24 h, after which luciferase activity in cell extracts was determined following the manufacturer's instructions. Data were expressed as a ratio of IL-10 promoter-driven luciferase activity divided by the renilla luciferase activity. Transfection with the empty reporter vector (pGL2B or pTAL) yielded no detectable activity.

### CREB Silencing Using siRNA

RAW264.7 macrophages were grown for 24 h in 6-well plates, at 6·10^5^ cells per well, in culture medium supplemented with 10% FBS. Transfection with siRNA against CREB (or a scrambled control sequence) was performed as described by Fraser et al. (36). A mixture of each siRNA with Lipofectamine2000 transfection reagent, initially incubated in OptiMEM medium for 20 min at room temperature, was added to the cells at 100 nM for the first 4 h, after which the volume was increased so the siRNA was at a concentration of 62.5 nM for the following 20 h. The cells were washed and the transfection process was repeated the next day for another 24 h. The siRNA-containing medium was removed and the cells were seeded in a 48-wells plate for a recovery period of 24 h. LPS (100 ng/ml) ± isoproterenol (1 μM) were then added for 4 h at 37°C. CREB expression was analyzed by western blotting and IL-10 production by ELISA.

### Construction of Plasmids

The full IL-10 promoter (−1,538/+64) luciferase reporter plasmid was mutated at the CREs and/or Sp1 sites according to the Quickchange^TM^ standard protocol (37). The sense primers for mutagenesis are listed below:

CRE1 - 5′-TAGCCCATTTATCCACaaaATTATGACCTGGGAGTGCG-3′,
CRE2 - 5′-CGTCATTATGACCTGGGAGTaaaTGAATGGAATCCAC-3′,
Sp1 - 5′-GGTTTAGAAGAGGGAGGAaaAGCCTGAATAAC-3′.

The heterologous reporter constructs: CRE1x4 (TTTATCCACGTCATTATG), CRE2x4 (GGGAGTGCGTGAATGGA), CRE consensus x4 (GGGAGTGACGTCAATGGA), IL-10 promoter Sp1 site x4 (GGAGGAGGAGCC) carrying four copies of the respective cis element upstream to a luciferase reporter gene, and the CRE1+CRE2 heterologous reporter carrying two copies of the IL-10 promoter region encompassing both CRE1 and CRE2 (-362/-323 relative to TSS), were generated using double stranded pre-synthesized oligonucleotides (Hylabs, Israel) cloned into the pTAL vector (Clontech, CA). The shRNA vector against IFNαR1 was constructed by cloning the shRNA oligonucleotide sequence

(GATCGGAATGAGGTTGATCCGTTTATCTCGAGATAAACGGATCAACCTCATTCTTTTTG) into the pGFP-RS shRNA vector (Origene, Rockville, MD). Sequence verification was performed using the ABI PRISM 3100 Genetic Analyzer sequencer. Plasmid production was done using Endofree Plasmid Maxi Kit.

### Transcription Factor-DNA Interaction Assay

We used QPID to measure TFs affinity to a library of DNA sequences derived from the mouse IL-10 promoter (38). A microfluidic device was designed and fabricated as described by Maerkl and Quake (39). The device was aligned to a dilution series microarray of Cy5-labeled dsDNA sequences (see Table 1) and its surface was derivatized as previously described (41–43). A construct of CREB tagged with both His_6_ and c-Myc, and a construct of ATF1 tagged with both HA and V5, were prepared and proteins expressed *in-vitro* as previously described (41). Homo- and hetero-dimers of CREB and ATF1 were introduced into the microfluidic device, and spotted DNA was solubilized, allowing interaction with the transcription factors. Mechanically induced trapping of molecular interactions (MITOMI) was performed after 1 h incubation (39) to enable quantification of each interacting component (41). Data were fitted and K_d_ values determined using non-linear least squares minimization. Binding experiments for some sequences were repeated with AP-1 dimers, composed of doubly tagged c-Fos, c-Jun, and ATF2.

**Table 1:** K_d_ values for binding of CREB and AP-1 heterodimers to CRE sequences.

**Oligo**	**Position**^a^		**Sequence**		**Kd (μM) for various TF dimers**				
	**Site**	**Context**			**CREB/ATF1**	**CREB**	**ATF1**	**c-Jun/c-Fos**	**c-Jun/ATF2**
CRE^b^	–	−349/−315	consensus	**TGACGTCA**	0.06	0.07	0.03	0.15	0.05
CRE1	−357/−350	−368/−336	wt	CC**ACGTCA**	0.06	0.56	0.05	3	2
			mutant	CC**A**aaaat	84	47	32		
CRE2	−335/−329	−349/−315	wt	**TG**-**CGT**G**A**	13	66	16	n.d.^c^	n.d.^c^
			mutant	**T**a-aa**T**G**A**	n.d.^c^	109	48		
CRE1+ CRE2	As above	−366/−298	wt	As above	0.05	1.6	0.08		
			mut CRE1	As above	10	61	10		
TRE^b^	–	−349/−315	consensus	**TGA**-**GTCA**	12	53	17	0.5	9

a *Position in the mouse IL-10 promoter, relative to TSS. Site = cis element, context = promoter region present in the oligonucleotide used for the binding assay*.

b *The depicted consensus sequence (40) was inserted in the context of the IL-10 promoter −349/−315 oligonucleotide, replacing the CRE2 sequence. Similar results were obtained when it was inserted in the context of the TNFα promoter CRE region*.

c *n.d., no binding detected*.

### Protein Determination

Protein was determined by a modification of the Bradford procedure, which yields linear and thus more accurate results, increased sensitivity, and reduced detergent interference, as previously described by Zor and Selinger (44) and Ernst and Zor (45). BSA served as standard.

### Western Blot Analysis

Whole cell lysates were prepared and used for western blot assays of CREB as previously described (17). Two-color imaging and quantitative analysis of western blots was performed using the Odyssey infrared imaging system (Li-Cor Biosciences), according to the manufacturer's instructions. Signal intensity was verified to be linear with protein quantity. An antibody against α-tubulin served for normalization.

### Statistical Analysis

Data were analyzed using one- or two-ways ANOVA with the appropriate multiple comparison test wherever applicable, as indicated in the figure legend. In all cases, differences of *p* < 0.05 were considered to be significant. All experiments were repeated at least twice.

## Results

### The Autocrine Type I IFN Loop Confers cAMP-Insensitive LPS-Stimulated IL-10 Expression at the Late Phase

**Figure 1:**
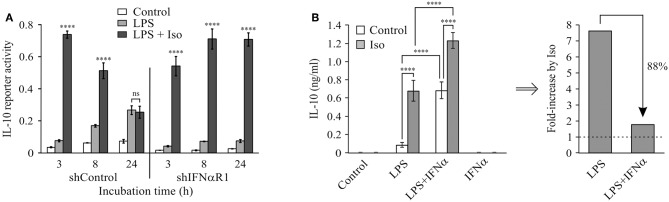
Type I IFN signaling dominates late LPS-stimulated IL-10 expression and hinders a synergistic effect of cAMP. **(A)** Silencing of the type I IFN receptor enables synergistic IL-10 expression by LPS and isoproterenol also at the late phase. RAW264.7 macrophages were transfected with a full mouse IL-10 promoter reporter construct, and with a plasmid encoding shRNA against either IFNαR1 or a control sequence. The cells were incubated with LPS (10 ng/ml) ± isoproterenol (Iso, 1 μM) for the indicated time. Luciferase reporter data represent three independent experiments and are expressed as mean ± SD of values normalized against renilla luciferase activity; ****p < 0.0001 compared to cells treated with LPS alone (two-way ANOVA followed by Tukey's multiple comparison test). The experiment was carried out three times with similar results. **(B)** Isoproterenol and IFNα exclusively synergize with LPS in IL-10 expression. Mouse macrophage RAW264.7 cells were incubated with LPS (10 ng/ml) and/or isoproterenol (Iso, 1 μM) and/or IFNα (1,000 units/ml) for 3 h. IL-10 secretion to the medium was measured by ELISA and data representing six independent experiments are expressed as mean ± SD; ****p < 0.0001 (two-way ANOVA followed by Tukey's *post-test*). IL-10 secretion from cells that were not treated with LPS was undetectable (<40 pg/ml). The experiment was carried out 3 times with similar results. Right panel—fold-increase by isoproterenol, calculated from the data presented in the left panel.

We recently demonstrated that elevated intra-cellular cAMP synergizes with LPS at IL-10 expression and secretion in LPS-stimulated RAW264.7 macrophages only at the early (3 h), but not late (24 h), phase (11). The temporal regulation trend of IL-10 protein expression was recapitulated in primary macrophages (BMDM), as well as *in-vivo* and was demonstrated also at the mRNA expression level in macrophages (11). We further showed that the loss of cAMP effect at the late phase was specific to IL-10 expression, while general cAMP-dependent transcriptional activity was retained (11). In contrast, autocrine/paracrine type I IFN activity is required for LPS-stimulated IL-10 expression at the late phase (2–7). We showed that neither recombinant IFNα nor secreted type I IFNs (conditioned medium from LPS-stimulated macrophages) can synergize with cAMP in IL-10 promoter activation (11). In the present study we further examined the interplay between type I IFN and cAMP in time-dependent IL-10 expression by silencing the common type I IFN receptor subunit, IFNαR1. To this end, RAW264.7 macrophages were co-transfected with a shIFNαR1 plasmid together with the IL-10 promoter reporter plasmid. Consistently with our previous report (11), the β-AR agonist isoproterenol, which stimulates intra-cellular cAMP formation (17), synergistically elevated LPS-stimulated IL-10 promoter reporter activity in control cells at the early phase, but not at the late phase (Figure 1A). Silencing the type I IFN receptor significantly reduced LPS-stimulated and basal IL-10 promoter reporter activity in a time-dependent manner, and strikingly—enabled synergism between LPS and isoproterenol during the entire 24 h time-course (Figure 1A). This dramatic effect of IFNαR1 silencing, taken together with the inability of exogenous IFNα to synergize with isoproterenol (11), suggests that normally the cAMP pathway can amplify only the low-direct IL-10 inducing effect of LPS at the early phase, whereas an autocrine IFNαR1-dependent activity which cannot cooperate with the cAMP pathway dominates late IL-10 induction in LPS-stimulated macrophages.

We further examined how LPS-dependent IL-10 expression is stimulated by isoproterenol vs. type I IFN by incubating RAW264.7 macrophages with various combinations of LPS, isoproterenol and IFNα for 3 h. LPS alone only slightly stimulated IL-10 expression and secretion at this early time frame, while isoproterenol alone and IFNα alone had no detectible effect. Yet, either isoproterenol or IFNα synergistically amplified LPS-dependent IL-10 secretion by nearly 8-fold (Figure 1B). Importantly, the effect of isoproterenol on LPS-induced IL-10 expression was reduced in the presence of IFNα by 88%—from 7.7-fold in cells treated by LPS, to 1.8-fold in cells co-treated by LPS and IFNα together (Figure 1B). The usage of IFNβ rather than IFNα similarly resulted in synergism with LPS and diminished amplification by cAMP (not shown). These findings indicate that LPS-dependent IL-10 expression can be synergistically amplified by either the cAMP pathway or type I IFN signaling, but in a largely exclusive manner. Both stimuli act permissively, i.e., inducing IL-10 expression only in macrophages co-treated with LPS. Moreover, even the combination of isoproterenol and IFNα (Figure 1B) or IFNβ (not shown) was incapable of inducing IL-10 expression in the absence of LPS. Together with the lack of additivity of their synergistic potentials, this suggests that cAMP signaling and type I IFN signaling affect a common step in IL-10 expression.

### CREB Is Required for Transcriptional Activation by cAMP at the −376/−295 bp Region of the Mouse IL-10 Promoter

**Figure 2:**
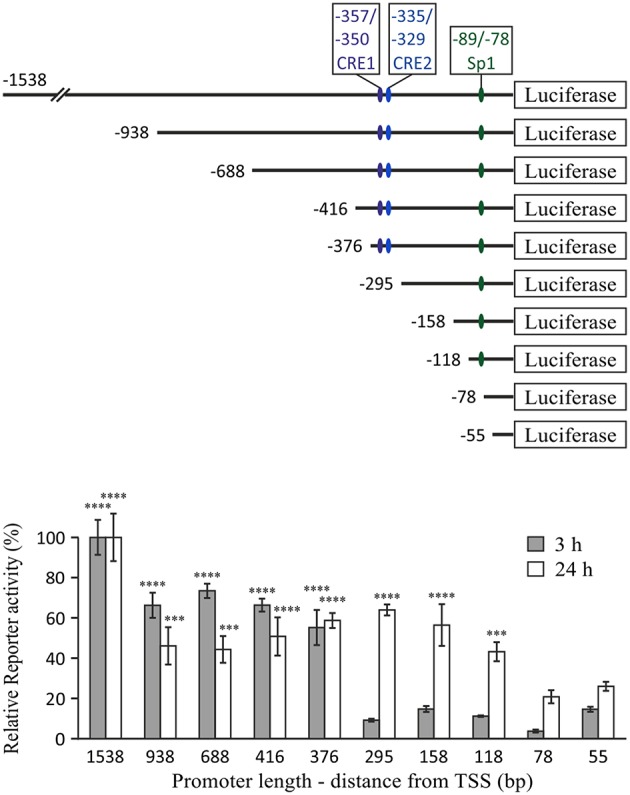
The −376/−295 bp region of the mouse IL-10 promoter mediates the early synergistic effect of cAMP. RAW264.7 macrophages, transfected with either of the indicated mouse IL-10 promoter deletion mutant reporter constructs, were incubated with LPS (10 ng/ml) and isoproterenol (Iso, 1 μM) for 3 or 24 h. Luciferase reporter data represent three independent experiments and are expressed as mean ± SD of values normalized against renilla luciferase activity, divided by unstimulated control cells and relative (in %) to full (1,538 bp) promoter reporter activity (for which stimulation by LPS+Iso was 35.5-fold and 40-fold, relative to resting cells, at 3 h and 24 h, respectively); ***p < 0.001, ****p < 0.0001 for cells transfected with the indicated deletion mutants compared to cells transfected with the −295 bp (3 h) or −78 bp (24 h) promoter reporter (two-way ANOVA followed by Dunnett's *post-test*). The experiment was carried out 3 times.

IL-10 mRNA and protein expression regulation by cAMP in LPS-stimulated cells was most sensitively reflected in direct up-regulation of transcription, as measured using an IL-10 promoter reporter (11). Induction of IL-10 promoter activity by cAMP elevation was minimal, unless the macrophages were co-stimulated by LPS (11). Thus, in the current study we set a goal to identify the promoter region accountable for the synergistic IL-10 inducing effect of the cAMP elevating agent isoproterenol in LPS-stimulated macrophages, using a series of 5′ deletion mutants of the mouse IL-10 promoter reporter (26). We reasoned that cAMP sensitivity will be manifested by identifying a promoter region critical for IL-10 reporter induction by a co-stimulus of LPS and isoproterenol only at the early phase. We indeed found that the promoter region at −376/−295 bp is most critical only during the early phase (3 h) of LPS and isoproterenol co-treatment (Figure 2). In contrast, at the late stage (24 h) of co-stimulation, the −376/−295 bp region was irrelevant while the −118/−78 bp region was important (Figure 2), as previously reported for 24 h of stimulation by LPS alone (26). The −1,538/−938 bp region was found to contribute to IL-10 expression at both early and late stages (Figure 2). These results suggest that early IL-10 transcription critically depends on a cAMP-regulated TF binding site located between 295 and 376 bp upstream of the TSS, and that LPS-regulated response elements located at the −118/−78 and −1,538/−938 bp regions are the dominant regulatory sites of IL-10 transcription at the late phase. We further focused on the −376/−295 bp region, as it was the only region demonstrating time-dependent relevance that fully matched the time course of regulation by cAMP on IL-10 promoter reporter activation (Figure 1A) and endogenous IL-10 expression (11).

**Figure 3:**
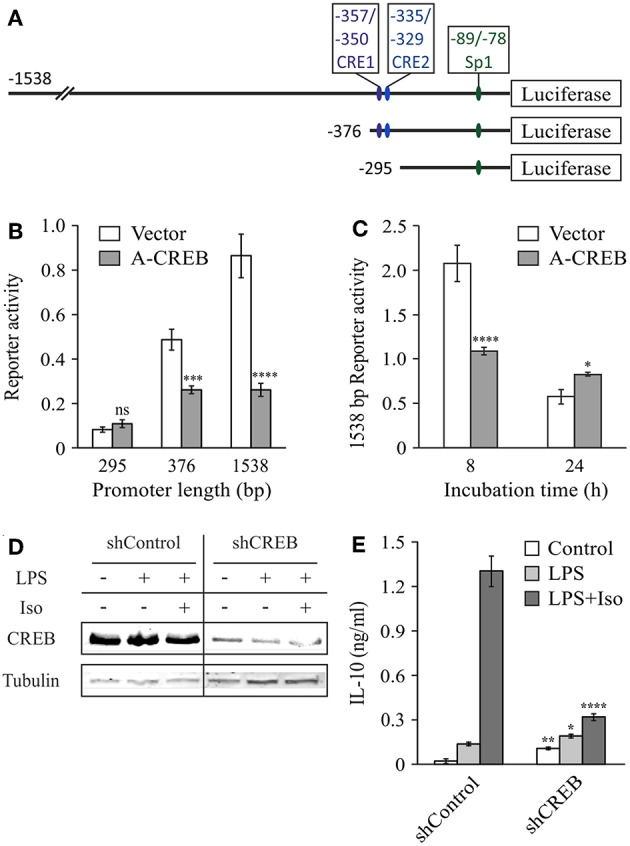
CREB mediates the early synergistic effect of cAMP. **(A–C)** Dominant negative CREB (A-CREB) inhibits mouse IL-10 promoter activity via the −376/−295 bp region. RAW264.7 cells were transfected with the indicated **(B)** or with the full (1,538 bp) **(C)** IL-10 promoter reporter plasmids and with A-CREB, or its control vector. The cells were treated with LPS (10 ng/ml) and isoproterenol (Iso, 1 μM) for 3 h **(B)**, 8 h or 24 h **(C)**. Luciferase reporter data represent three independent experiments and are expressed as mean ± SD of values normalized against renilla luciferase activity; *p = 0.015, ***p = 0.0002, ****p < 0.0001 for cells transfected with A-CREB compared to cells transfected with empty vector (two-way ANOVA followed by Sidak's *post-test*). The activity in resting cells was at least 3-fold lower than in treated cells. **(D,E)** CREB silencing blocks isoproterenol-stimulated IL-10 expression in LPS-treated macrophages. Stably CREB-silenced and shRNA-control RAW264.7 cells were incubated with LPS (10 ng/ml) ± isoproterenol (Iso, 1 μM) for 3 h. **(D)** CREB levels were analyzed by western blot. **(E)** IL-10 secretion to the medium was measured by ELISA and data representing eight independent experiments are expressed as mean ± SD; *p = 0.025, **p = 0.002, ****p < 0.0001 for silenced cells compared to control cells (two-way ANOVA followed by Sidak's *post-test*). All experiments were carried out twice with similar results.

To explore the role of CREB in IL-10 promoter activation and to confirm the location of the cAMP-sensitive region (Figure 3A), we co-stimulated the cells with LPS and isoproterenol and used the dominant negative construct A-CREB, which sequesters native CREB by dimer formation and is unable to bind the DNA (34). Figure 3B shows that at 3 h, A-CREB inhibits reporter activity of the full 1,538 bp IL-10 promoter as well as of the shorter 376 bp construct, but has no effect on transcriptional activation of the further-shortened 295 bp IL-10 promoter. A-CREB also inhibits co-stimulation of the full (1,538 bp) IL-10 promoter reporter at 8 h but has no negative effect at 24 h (Figure 3C). These results support the finding above regarding the location of the cAMP-regulated site at the −376/−295 bp region of the mouse IL-10 promoter, and suggest that CREB mediates the enhancing effect of isoproterenol on IL-10 reporter activity at the early (and mid-) phase whereas late IL-10 expression in LPS-stimulated cells is CREB-independent. Next, we validated the involvement of CREB in the regulation of endogenous IL-10 expression by the cAMP pathway, using a previously described RAW264.7 cell line (30) that stably expresses shRNA against CREB1a (hereafter shCREB), resulting in 80% CREB silencing efficiency (compared to shControl cells, Figure 3D). As observed with the dominant negative approach, isoproterenol was unable to significantly stimulate LPS-induced IL-10 secretion at the early phase in shCREB cells, unlike control cells (Figure 3E). Furthermore, transient siRNA-mediated CREB silencing (95% efficiency at the protein level) diminished the synergistic effect of isoproterenol (data not shown). These results indicate that CREB mediates the synergistic effect of cAMP on IL-10 expression in LPS-stimulated macrophages, and is consistent with a previous report, in which IL-10 mRNA levels in shCREB cells stimulated with LPS for 2 h were not further increased by cell-permeable cAMP (30).

### Cooperative Tandem CRE Sites at the Mouse IL-10 Promoter

The human IL-10 promoter contains a single CRE which was demonstrated to be functional upon stimulation with exogenous cAMP for 24 h and is also conserved in the mouse IL-10 promoter (31). This site resides at −357/−350 bp relative to the TSS (hereafter CRE1, Table 1), within the region identified in Figure 2. Mutation of that conserved cis element at the human promoter only partially reduced the response to a cAMP stimulus (31), and thus we decided to perform a bioinformatics search to identify additional putative CREs within the −376/−295 bp region. A putative CRE-like 7 bp sequence was indeed found 21 bp apart from CRE1 (3′ to 3′), at −335/−329 bp relative to the TSS (hereafter CRE2, Table 1).

To assess the binding of these sequences to CREB and its closely-related family member ATF1, we used a microfluidics approach named Quantitative Protein Interactions with DNA (QPID) (38, 39). We spotted increasing concentrations of Cy5-labeled oligonucleotides on a microfluidic array device; CREB and ATF1 homo- and hetero-dimers were allowed to bind and reach equilibrium, and we then quantified the protein-DNA interaction via fluorescence of the tags present on the DNA and the TF-bound antibodies. As shown in Table 1, the CREB homodimer bound to a CRE1 oligonucleotide with an affinity that was one order of magnitude lower than to a consensus CRE sequence, but two orders of magnitude higher than to a CRE2 oligonucleotide. CREB binding to an oligonucleotide containing both CRE1 and CRE2 was comparable to CRE1 alone. The binding affinities of the CREB homodimer to CRE1 and CRE2 were an order of magnitude lower than those of a ATF1 homodimer or CREB-ATF1 heterodimer. Compared to the CREB family members, AP-1 heterodimers displayed comparable high affinity to consensus CRE, low affinity to CRE1 and non-detected binding to CRE2. Based on these results, we predicted that CRE1 would be activated by cAMP-stimulated CREB/ATF-1, but not by LPS which stimulates AP-1 activity (40).

**Figure 4:**
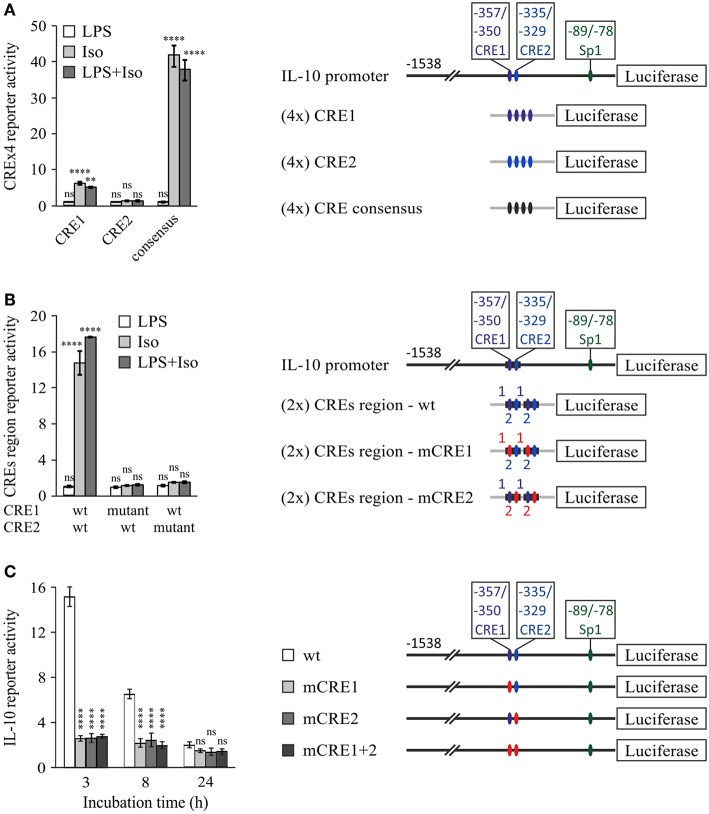
Cooperativity between tandem CRE sites is essential for cAMP-stimulated IL-10 promoter activity. RAW264.7 macrophages were transfected with a luciferase reporter regulated by four repeats of either CRE1 or CRE2 or a consensus CRE **(A)**, two repeats of the −362/−324 bp region with either the WT sequence, CRE1 mutant, or CRE2 mutant **(B)**, or the full (−1,538 bp) IL-10 promoter, either wt, mutant in CRE1 (mCRE1), mutant in CRE2 (mCRE2) or a double mutant (mCRE1+2) **(C)**. Site mutation is shown by red color. The cells were incubated with isoproterenol (Iso, 1 μM) and/or LPS (10 ng/ml) for 3 h **(A,B)** or with both stimuli for the indicated time **(C)**. Luciferase reporter data represent three independent experiments and are expressed as mean ± SD of values normalized against renilla luciferase activity, relative to unstimulated **(A,B)** or LPS-stimulated **(C)** control cells; **(A,B)** **p = 0.001, ****p < 0.0001 for cells treated with isoproterenol (± LPS) compared to control cells (two-way ANOVA followed by Dunnett's *post-test*). **(C)** Error bars represent the sum of SD (%) of both values in the ratio. ****p < 0.0001 for mutants compared to wt (two-way ANOVA followed by Dunnett's *post-test*). The experiments were carried out 3 times with similar results.

To examine the potential of the CRE1 and CRE2 sequences to mediate CREB-dependent transcription, we constructed reporter plasmids carrying four repeats of either sequence and compared their activity to that of the consensus CRE. Figure 4A shows that the CRE1 construct was activated 6-fold by isoproterenol in a 3 h assay, whereas the CRE2 construct was not activated, and the CRE consensus sequence was activated 43-fold by isoproterenol. Notably, CRE1 contains one consensus position in addition to a consensus CRE half-site (5 bp) which is known to be weakly activated by CREB relative to the full 8 bp palindrome CRE (46). In contrast to isoproterenol, LPS neither activated these sequences by itself nor affected the activity of isoproterenol (Figure 4A). Next, we created reporter plasmids regulated by two repeats of the entire −362/−324 bp region of the IL-10 promoter containing both CRE1 and CRE2, wild-type (wt) or mutated in either sequence. Surprisingly, the 16-fold reporter activation induced by isoproterenol was not only completely abolished by mutation of CRE1 but also completely abolished by mutation of CRE2, indicating tight cooperativity between the two CRE sequences (Figure 4B). Finally, we created CRE mutants of the full IL-10 promoter reporter. These mutations only modestly affected the low LPS-induced activity at 3 h, and thus, in order to focus on the relevance of each CRE sequence to IL-10 transcription induction by cAMP, the Y axis in Figure 4C depicts IL-10 reporter activities in cells stimulated by LPS and isoproterenol, relative to LPS alone. Mutation of either CRE1 or CRE2 sharply reduced isoproterenol's effect on LPS-induced IL-10 reporter activity at the early and mid- phases (Figure 4C). Importantly, mutation of both CRE1 and CRE2 in the context of the full IL-10 promoter was just as detrimental as mutation of only a single CRE (Figure 4C). Consistent with the previous experiments, isoproterenol's effect on LPS-induced IL-10 reporter activity was time-dependent (Figure 4C). Thus, these results indicate complete synergism between CRE1 and CRE2 in mediating amplification of early LPS-induced IL-10 transcription in response to a cAMP stimulus.

### Sp1 Critically Regulates IL-10 Transcription in Cooperativity With CRE

**Figure 5:**
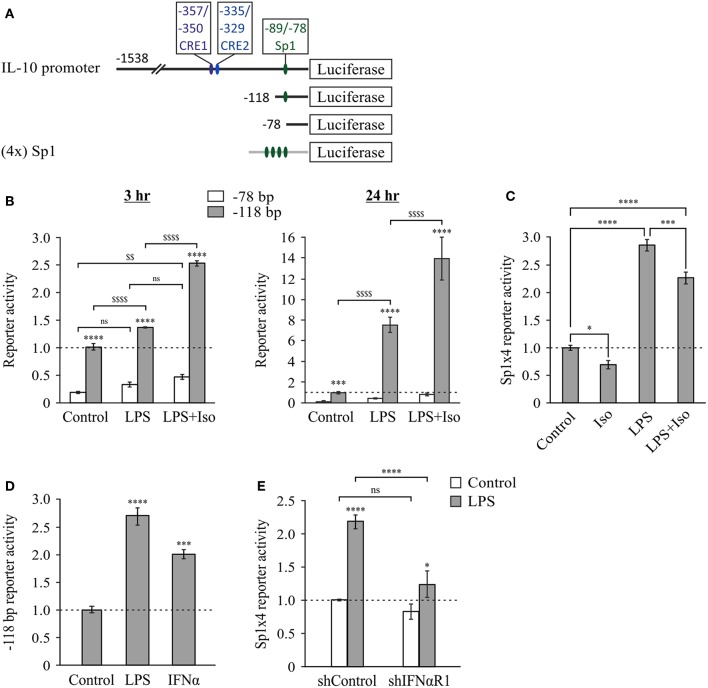
The Sp1 response element at −89/−78 bp is constitutive at the early phase and further activated by LPS via type I IFN at the late phase. **(A)** Plasmid constructs used in the following panels. RAW264.7 cells were transfected with either the −78 bp **(B)** or the −118 bp **(B,D)** 5′-deletion IL-10 promoter reporter plasmid, or with a reporter construct regulated by four repeats of the putative IL-10 promoter Sp1 sequence (−89/−78 bp) **(C,E)**. **(E)** The cells were co-transfected with a plasmid encoding shRNA against either IFNαR1 or a control sequence. **(B–E)** The cells were incubated with vehicle, LPS (10 ng/ml) and/or isoproterenol (Iso, 1 μM) or with mouse IFNα (1,000 units/ml), for 3 h (**B**—left panel) or 24 h (**B—**right panel, and **C–E**). Luciferase Reporter data represent three independent experiments and are expressed as mean ± SD of values normalized against renilla luciferase activity and relative to unstimulated control cells. **(B)** ****p < 0.0001 for cells transfected with −118 bp mutant compared to the −78 bp mutant (two-way ANOVA followed by Sidak's *post-test*); ***p = 0.0004 for resting cells transfected with −118 bp mutant compared to the −78 bp mutant (Student's *t*-test); ^$$^*p* = 0.008, ^$$$$^*p* < 0.0001 for cells transfected with the same plasmid and treated with different stimuli (two-way ANOVA followed by Sidak's *post-test*). All values were above the detection limit, except for resting control cells transfected with the −78 bp mutant. **(C)** *p = 0.01, ***p = 0.001, ****p < 0.0001 (one-way ANOVA followed by Sidak's post-test). **(D)** ***p = 0.001, ****p < 0.0001 (one-way ANOVA followed by Dunnett's *post-test*). **(E)** *p = 0.025, ****p < 0.0001 (two-way ANOVA followed by Sidak's *post-test*). The experiments were carried out 3 times **(B)**, 6 times **(C)** or twice **(D,E)** with similar results.

The Sp1 site located at −89/−78 bp was shown to mediate transcriptional induction of the mouse IL-10 promoter in macrophages stimulated with LPS for 24 h (26). However, its role at shorter LPS stimulation periods and its relevance regarding synergistic IL-10 expression have not been reported. Therefore, we initially compared the LPS-inducible activities of 5′-deletion constructs containing (−118 bp) or not containing (−78 bp) the reported Sp1 binding site (Figure 5A). As shown above (Figure 2), the activity of the −118 bp reporter in stimulated cells relative to resting cells was surprisingly similar to that of the shorter reporters at 3 h and only modestly higher (~2-fold) for the Sp1-containing construct at 24 h. However, separate analysis of the activities in resting cells, LPS-stimulated cells and cells co-stimulated by LPS and isoproterenol, indicated that deletion of the region that includes the Sp1 loci greatly reduces IL-10 promoter activity in all cellular activation states at both 3 h and 24 h (Figure 5B). These results imply that Sp1 has a critical role in both basal and inducible transcription of IL-10. Importantly, while LPS only slightly elevated the activities of the −78 and −118 bp reporters at the early phase (Figure 5B, left panel), it greatly stimulated the late phase activity of both reporters, and in particular that of the promoter construct that contains the Sp1 response element (−118 bp)—by an order of magnitude (Figure 5B, right panel). The modest positive effect of isoproterenol on LPS-stimulated activity of the −118 bp promoter was significantly less pronounced than that of LPS (Figure 5B, right panel), and isoproterenol had no effect on the basal activity of the 5′-deletion constructs or the full IL-10 promoter reporter in resting cells (not shown). These results suggest that at long incubations LPS up-regulates the activities of Sp1 and of another TF binding downstream to −78 bp, likely to be NFκB p50 homodimer, as we (47) and others (29) have reported.

To further examine the ability of LPS to activate IL-10 transcription via the Sp1 site, we prepared a reporter plasmid regulated by four repeats of the IL-10 promoter Sp1 site. Consistent with the above findings, LPS was unable to induce reporter activity at the early phase (not shown), but stimulated its activity 3-fold at the late phase (24 h) (Figure 5C). In contrast, isoproterenol was unable to increase, and even partially decreased, both the basal activity and the LPS-stimulated activity of the reporter (Figure 5C). The relatively modest effect of LPS on the Sp1 reporter suggests that in the full IL-10 promoter the Sp1 site cooperates with additional cis elements. Alternatively, different spacing between the four Sp1 sites in the reporter may enable a higher response to LPS. Nevertheless, our results indicate that LPS, but not isoproterenol, stimulates Sp1 activity at the late phase.

The opposite time-dependency of CREs activation by cAMP and Sp1 activation by LPS (early vs. late, respectively), echo the time-dependencies of IL-10 expression stimulation by cAMP and autocrine type I IFN (11) (Figure 1A). We therefore examined whether type I IFNs are involved in LPS-stimulated activation of the Sp1 response element of the IL-10 promoter. This indeed was demonstrated by the following two experiments. First, the minimal IL-10 promoter reporter that includes the Sp1 site (−118 bp) was activated by a 24 h treatment with either LPS or IFNα (Figure 5D). The lower reporter stimulation by IFNα, relative to LPS, suggests that the autocrine type I IFN loop is required, but not sufficient for maximal Sp1 activation in response to LPS, as we showed also for the reporter of the full IL-10 promoter (11). Second, silencing the common type I IFN receptor subunit, IFNαR1, almost completely abolished LPS-stimulated activity (at 24 h) of the reporter for the Sp1 response element from the IL-10 promoter (Figure 5E). Taken together, our results suggest that LPS up-regulates IL-10 transcription at the late phase, at least in part via autocrine type I IFN stimulating Sp1 activity at the −89/−78 bp cis element.

**Figure 6:**
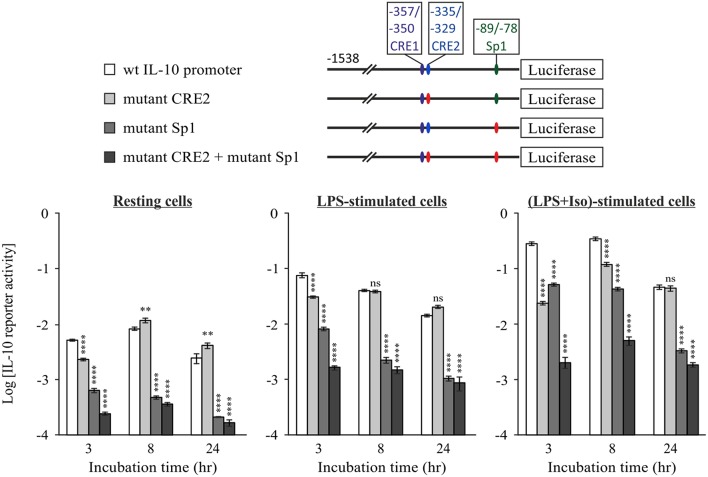
The constitutive Sp1 response element cooperates with cAMP-stimulated CRE at the early phase, but not at the late cAMP-insensitive phase. RAW264.7 cells were transfected with the indicated WT or mutant full IL-10 promoter reporter plasmids. Site mutation is shown by red color. Reporter activities were measured following 3–24 h (as indicated) of treatment with vehicle **(left panel)** or with LPS (10 ng/ml) in the absence **(middle panel)** or presence **(right panel)** of isoproterenol (Iso, 1 μM). Luciferase Reporter data represent three independent experiments and are expressed as mean ± SD of values normalized against renilla luciferase activity. **p < 0.01, ****p < 0.0001 for cells transfected with a mutant reporter compared to WT IL-10 promoter reporter (two-way ANOVA followed by Dunnett's *post-test*). The experiment was carried out twice with similar results.

**Figure 7:**
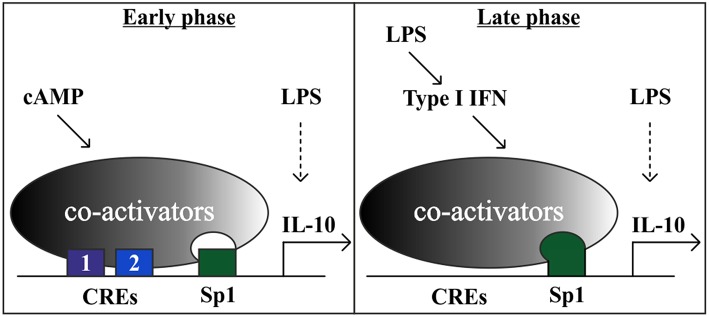
A model showing that the cAMP pathway accelerates IL-10 transcription in LPS-stimulated macrophages at the early phase via a tandem set of CREs that synergize with a constitutive Sp1 site. At the late phase, the strong LPS-induced IL-10 transcription is mediated in part by an autocrine type I IFN loop, which relies on hyper-activation of the Sp1 site and obviates the requirement for the cAMP pathway. Additional LPS-regulated TFs, that are not depicted, include for example p50 NFκB homodimer (47) and STAT1/3 (3).

To examine the role of the Sp1 response element in the context of the full IL-10 promoter and in relation to the CRE sites, we mutated the Sp1 sequence in the full mouse IL-10 promoter reporter, alone or together with mutation of the CRE2 sequence. Figure 6 shows WT and mutant IL-10 promoter reporter activities at 3, 8, and 24 h, in resting cells (left panel) and in cells stimulated with LPS in the absence or presence of isoproterenol (middle and right panels, respectively). Note that the Y axis has a logarithmic scale. Mutation of the Sp1 site resulted in a loss of activity by at least an order of magnitude at all incubation periods in resting and stimulated conditions. The detrimental effect of substitution mutation (Figure 6) or deletion (Figure 5B) of the Sp1 response element implies a critical role for that TF in IL-10 expression. Mutation at the CRE2 site reduced IL-10 reporter activity in cells co-stimulated with LPS and isoproterenol by an order of magnitude at 3 h, but had a more modest effect at 8 h and no effect at 24 h. The CRE2 mutation only moderately affected IL-10 reporter activity in both resting cells and cells stimulated with LPS alone for 3 h, and had no effect on IL-10 reporter activity during the longer LPS incubations of 8 and 24 h. This may represent a modest contribution of basal cAMP levels to early LPS-stimulated IL-10 expression, or a modest role for autocrine LPS-induced factors which elevate cAMP, such as eicosanoids (48), or a small medium replacement artifact (35). In any case, the effect of Sp1 mutation is considerably more pronounced than that of CRE2 mutation in all cellular states and time points, except for 3 h of co-stimulation with LPS and isoproterenol. Finally, while mutation of either CRE2 or Sp1 reduces IL-10 reporter activity in response to 3 h of co-stimulation (LPS and isoproterenol) to 8.5 and 18.1%, respectively, of WT IL-10 reporter activity, mutation of both CRE2 and Sp1 together reduces the respective IL-10 reporter activity to only 0.7% of WT activity (Figure 6, right panel). This synergism between the two cis elements is also evident at 8 h. Taken together, our results suggest (see cartoon in Figure 7) that cAMP-elevating agents up-regulate early (3 h) LPS-induced IL-10 expression by transcriptional activation at the CRE sites, which cooperate with the constitutive Sp1 site. At 24 h, the role of the Sp1 site is strengthened due to its activation by the LPS pathway via an autocrine type I IFN loop, whereas the CRE sites become largely irrelevant.

## Discussion

### Crosstalk Between the cAMP Pathway and Type I IFN Signaling Regarding IL-10 Expression in Macrophages

Anti-inflammatory macrophages, characterized by reduced production of pro-inflammatory cytokines and increased levels of IL-10, mediate inflammation resolution and homeostasis. While these macrophages usually appear at a late stage of LPS stimulation, such macrophage sub-populations can also be generated following co-stimulation by a TLR ligand and a second stimulus, including an IgG immune complex, apoptotic cell remnants, or a cAMP inducer (1).

We have previously shown that elevation of cAMP stimulates IL-10 expression in mouse macrophages in synergism with LPS (9), which occurs only at the early, but not late, phase of LPS stimulation (11). Mechanistically, we ruled out receptor desensitization as a possible explanation for the loss of synergism (11), and instead suggested that autocrine type I IFN signaling which is required for IL-10 expression at the late phase of LPS stimulation (2–7) interferes with cAMP effect at late IL-10 expression. Indeed we demonstrate that type I IFN and cAMP amplify LPS-dependent IL-10 expression by exclusive, non-additive and time-distinctive transcriptional mechanisms. This is concluded from a combination of evidences: (i) The inability of cAMP to synergistically elevate IL-10 expression with IFNα or with autocrine type I IFN present in conditioned medium from cells pre-treated with LPS for a prolonged time (11); (ii) The regained ability of cAMP to synergize with LPS even in late IL-10 reporter expression upon type I IFN receptor silencing; (iii) The diminished ability of cAMP to synergistically elevate early IL-10 secretion in cells treated with a combination of LPS and IFNα; (iv) The opposing time-dependencies of the different IL-10 promoter sites activated by cAMP and type I IFN.

We show that an autocrine/paracrine type I IFN loop is essential for efficient activation of the IL-10 promoter in LPS-stimulated macrophages at the late stage, in accordance with previous reports (2–7). Yet, IFNα, similarly to cAMP inducers, can induce IL-10 expression only in the presence of LPS as a co-stimulator. This permissive property insinuates that in order to induce IL-10, both the LPS-driven autocrine type I IFN loop and the cAMP pathway must cooperate with a LPS-activated pathway(s) which is type I IFN-independent. Notably, LPS rapidly induces IL-10 mRNA expression without a concomitant activation of the IL-10 promoter reporter, pointing to IL-10 mRNA stabilization (11), which indeed was shown to occur via p38 (12, 13). Additionally, the ability of LPS to synergize with the cAMP pathway at the early phase, not only in endogenous IL-10 expression but also in IL-10 promoter reporter activation, suggests that LPS further augments cAMP-stimulated IL-10 transcription in a type I IFN-independent manner.

### Regulation of the Mouse IL-10 Promoter by the cAMP Pathway

In the current study we explored the molecular mechanism of the time-dependent synergism between cAMP and LPS using a set of mouse IL-10 promoter deletion mutants previously used by Brightbill et al. (26). In that study the reporter series was used to locate the Sp1 binding site at the mouse IL-10 promoter and to demonstrate its critical role in IL-10 expression in RAW264.7 macrophages stimulated for 24 h by LPS alone (26). We found that synergistic IL-10 promoter activation in the early response to LPS and cAMP requires, in addition to the Sp1 site, two proximate CRE sites located at −357/−350 and −335/−329 bp relative to the TSS. The CREs-dependent synergism is most pronounced at short (3 h) duration of co-stimulation by LPS and a cAMP-elevating agent and it is later reduced (8 h) and even abolished at the late phase (24 h). In contrast, Sp1 activity is constitutive at 3–8 h of LPS stimulation and is up-regulated only at 24 h. Basal activity at the CRE sites modestly contributes to the low LPS-stimulated IL-10 production in the absence of a cAMP inducer at the early phase (3 h). These results are consistent with studies done on IL-10 mRNA expression in BMDM derived from mice bearing a CREB S133A knock-in mutation, preventing phosphorylation (19, 32). The mutation reduced early (1 h), but not late (4 h) IL-10 mRNA expression in response to either LPS alone or to LPS and a cAMP elevating agent (19). Early IL-10 expression in the CREB S133A knock-in macrophages was reduced but still synergistic, likely due to ATF1 activation (19).

We confirmed the location of the cAMP-regulated region by using a dominant negative version of CREB that specifically inhibited the activity of only promoter constructs that include that region, but not of a shorter construct. A bioinformatics approach identified two putative CRE sites at that region, CRE1 and CRE2, whose critical role in mediating cAMP-dependent synergistic IL-10 transcription was established by point mutations. Surprisingly, the function of these adjacent CRE sites is completely inter-dependent. CRE1 and CRE2 have high and low affinities to CREB/ATF1 dimers, respectively, corresponding to their differential ability to mediate cAMP-dependent transcription from a heterologous promoter containing four copies of a single cis element. Interestingly, binding of the TF dimer to the weak CRE2 site does not increase the affinity of binding to the strong CRE1 site. It should be noted that the methodology used cannot exclude the opposite possibility that binding to the strong CRE1 site increases the affinity of binding to the weak CRE2 site. While our dominant negative approach blocks activity of both CREB and ATF1, the RNAi approaches specifically interfere with CREB and therefore suggest that CREB is more dominant than ATF1 in IL-10 induction, in line with a previous report (19). Consistently, there is a correlation between the magnitude of CRE1 and consensus CRE reporter activities in cells and their *in-vitro* binding affinities for CREB homodimers; yet, there is no such correlation with binding of ATF1-containing dimers. Taken together, these findings suggest that simultaneous binding of CREB dimers to the two adjacent CRE sites is required for synergistic IL-10 promoter activation at the early phase of co-stimulation by LPS and a cAMP-elevating agent.

As LPS activates the AP-1 transcription factor, which can potentially bind and activate CRE sites (40), it is important to note that the affinities of the two IL-10 promoter CRE sites for CREB/ATF1 are orders of magnitude higher than for AP-1 dimers and that these CREs were activated only by the cAMP pathway and not by LPS.

The two inter-dependent CRE sites are located within a 21 bp spacing (3′ to 3′ distance), which corresponds to two DNA helical turns. Therefore, the two CREB dimers are expected to be positioned closely and in parallel to each other, possibly interacting. This unique dual CRE arrangement also exists in the promoter of CREB itself, where two CRE half sites (TGACG) are distanced 21 bp apart from each other (3′ to 3′) and exhibit complete synergism (49), as we have shown for the IL-10 promoter CREs. Interestingly, in both the IL-10 and CREB promoters, the CRE sequences are imperfect, suggesting that the synergism between two 21 bp-spaced CRE sites results from or depends on relatively weak TF binding affinity to at least one of the sites. In contrast, the promoter of the human chorionic gonadotropin α-subunit is activated by cAMP via either of two perfect CRE consensus sequences that are distanced only 18 bp apart (3′ to 3′) and therefore the two CREB dimers are non-parallel, have high affinity to both sites, and act independently (50). Cooperative interactions with additional proteins may be the mechanism of synergism between the two CRE sites at the IL-10 promoter. The coactivators CBP/p300 (51) and CRTC/TORC (52, 53) are recruited by CREB and act in concert at promoters of CREB target genes (54). Thus, one can envision that the concurrent binding of two CREB dimers to the proximate CRE1 and CRE2 sites at the IL-10 promoter facilitates recruitment of the multiple coactivators required for subsequent promoter activation.

While CRE1 is conserved in sequence and location between mouse and human, the mouse IL-10 promoter CRE2, discovered in this study, is not conserved (31). Interestingly, the mouse CRE2 is positioned equivalently to an AP-2 response element at the human IL-10 promoter (31). AP-2 activation is achieved by integration of cAMP-PKA and PKC signaling (55). As LPS activates PKC (56) it can be inferred that a co-stimulus of LPS and a cAMP inducer activates the AP-2 response element at the human promoter. Thus, it is intriguing that although CRE2 is not conserved, a location similarly distanced from a conserved CRE has been conserved for a TF that relays cAMP signaling, either by itself (mouse, −335/−329 bp) or in combination with LPS signaling (human, −337/−328 bp).

### Regulation of the Mouse IL-10 Promoter by LPS

Brightbill et al. (26) showed that Sp1 is the major TF that mediates LPS-induced IL-10 transcription at 24 h and that it binds to a response element located at −89/−78 bp. We found that site to be regulated also in cells co-stimulated for 24 h with both LPS and a cAMP-elevating agent. Importantly, deletion of the −118/−78 bp region or site-directed mutagenesis markedly reduced IL-10 promoter reporter activities in both resting cells and stimulated cells. These results imply that Sp1 plays a critical role in both the basal transcription of IL-10 and in the inducible transcription caused by LPS and by LPS + isoproterenol, in line with a previous report (57). Consistently, Iyer et al. (3) found that the Sp1 site was critical for IL-10 reporter induction in LPS-stimulated RAW264.7 macrophages, but they showed that Sp1 is constitutively bound to the −89/−78 site at the chromosomal IL-10 promoter. Yet, we found that at 24 h LPS stimulated the activity of the promoter construct that contains the Sp1 response element (−118 bp) by an order of magnitude compared to resting cells, whereas a much smaller effect was observed for LPS at 3 h. This suggests that LPS significantly up-regulates Sp1 activity mainly at long incubations. Consistently, we showed that LPS stimulated expression of a reporter regulated by four copies of the IL-10 promoter Sp1 response element at 24 h, but not earlier. Furthermore, we show that IFNα also activates the minimal (−118 bp) promoter construct containing the Sp1 response element, and that autocrine type I IFN signaling mediates Sp1 reporter activation by LPS at the late phase. Taken together, the temporal correlation we found between Sp1 hyper-activation by LPS/type I IFN and loss of synergistic IL-10 induction by LPS and a cAMP-elevating agent insinuates that late Sp1 hyper-activation by LPS via type I IFN obviates the requirement for cAMP signaling to achieve maximal IL-10 transcription and expression.

The Sp1-mutant IL-10 promoter reporter was still induced at least 5-fold by LPS, suggesting that additional cis elements are involved in LPS-induced IL-10 transcription. Indeed, the short −78 bp reporter that lacks the Sp1 site was positively regulated by LPS at 24 h. Accordingly, over-expression of the NFκB p50 subunit in LPS-stimulated mouse macrophages amplified the activity of a short IL-10 promoter reporter that contains a specific p50-binding cis element located at −55/−46 bp in a CBP-dependent (29) and IκBζ-dependent (58) manner, while NFκB p50 knockout in mice diminished IL-10 secretion (29). Consistently, we have previously reported that selective inhibitors of NFκB p50 (but not p65) nuclear translocation blocked IL-10 secretion and reduced the activity of the short −78 bp IL-10 promoter reporter construct which contains the NFκB p50 homodimer response element, but does not contain the upstream Sp1 and CRE sites (47).

In the current study we also found that the −938 bp 5′-deletion mutant of the IL-10 promoter reporter has reduced LPS-dependent activity relative to the full −1,538 bp construct both at 3 h and 24 h, suggesting that the −938/−1,538 region includes a site regulated by LPS at both early and late phases. Indeed, Iyer et al. (3) showed that STAT3 and STAT1 mediate type-I IFN-dependent LPS-stimulated IL-10 expression via a cis element located at −1,324/−1,319.

While the reporter gene assay, and in particular the 5′-deletion approach, is useful for the study of gene regulation as demonstrated here, some regulatory sites may be overlooked. Examples include when a given cis element functions only in concert with another cis element (and so deletion of the distal site may prevent identification of the proximal site), when two cis elements are partially redundant, or when proximate cis elements are included in a single deletion. Furthermore, epigenetic regulation is also overlooked when using reporter constructs, as shown for immune complexes which synergize with LPS to induce chromosomal IL-10 expression but not IL-10 reporter (59). Nevertheless, we recently showed that the direct regulation of LPS-stimulated IL-10 promoter reporter activity by cAMP adequately reflects regulation of IL-10 mRNA and protein expression by this pathway (11).

### Mechanistic Basis for the Time-Dependency of Synergistic IL-10 Expression Amplification by cAMP

Both CRE and Sp1 mutations are detrimental for synergistic IL-10 reporter transcription at 3 h. The effect of Sp1 mutation is more pronounced in all other cellular states and time points. Early IL-10 expression by LPS alone depends mainly on Sp1 and to a lower extent on CRE, whereas during longer LPS stimulation, Sp1 activity becomes even more critical but CRE is irrelevant. Double mutation of the CRE and Sp1 sites reinforces the observations made by single mutations and highlights the synergism displayed between these two sites under all conditions where CRE is relevant. Together, these results imply that cAMP-elevating agents up-regulate LPS-induced IL-10 transcription at short (3 h) and medium (8 h) periods in a synergistic manner, via cooperativity of the cAMP-regulated CRE sites with the constitutive Sp1 site which is only later up-regulated by LPS via type I IFN. In support, CREB and Sp1 were reported to synergistically drive transcription at the Na,K-ATPase β1 promoter and to co-immunoprecipitate together with CBP (60). The interaction between CREB and Sp1 is likely to be indirect, as no direct binding was observed with recombinant proteins (61) and as both TFs independently bind CBP and TFIID (62). Moreover, the glutamine-rich domain of Sp1 can substitute for the equivalent Q2 domain of CREB in order to increase the DNA retention time governed by CREB's bZIP domain (63). Indeed, Zhang et al. (64) showed that cAMP induces activation of only a small and selective subset of the promoters which are occupied with phosphorylated CREB, and that this is reflected at the level of CBP recruitment which presumably depends on additional TFs to cooperate with CREB. We therefore propose that CREB and Sp1 synergize on the IL-10 promoter by stabilization of a complex involving these two TFs and co-activators (Figure 7). Notably, the Sp1 site appears to be mainly constitutive at the early phase, and therefore it is likely that additional LPS-regulated sites (such as those for p50 NFκB homodimer and STAT1/3) are involved in the synergistic expression of IL-10.

We showed here that the cAMP pathway specifically amplifies only the low type I IFN-independent IL-10 promoter activation by LPS that occurs at the early phase, while the strong IL-10 induction by LPS at the late phase is largely indirect (type I IFN-dependent) and not amenable for up-regulation by the cAMP pathway. In this study we also explored the mechanism of LPS-stimulated IL-10 expression via autocrine type I IFN, and showed that IFNα can partially mimic LPS in late Sp1 activation, and that type I IFN receptor silencing blocks activation of the IL-10 promoter region containing Sp1. Taken together with the findings of time-dependent cooperativity at the promoter level discussed above, our data suggest a model (Figure 7) where the cAMP pathway can synergize at the IL-10 promoter, via novel tandem CREs, with Sp1 acting at a constitutive level and with the TFs directly activated by LPS (e.g., p50 NFκB homodimer and STAT1/3), whereas the autocrine type I IFN loop dominates late LPS-stimulated IL-10 induction and prevents or obviates synergism with the cAMP pathway. Sp1 transcriptional activation by the autocrine type I IFN loop may explain, at least in part, the switch from synergistic IL-10 expression at the early phase to cAMP-insensitive IL-10 expression at the late phase. The time lag in IL-10 induction by LPS, resulting from the requirement for autocrine type I IFN, ensures a proportional inflammatory response to pathogen detection or tissue damage. However, certain pathogens manipulate the immune system to elevate IL-10 expression and reduce pro-inflammatory cytokine expression as a persistence mechanism (24). The accelerated induction of IL-10 when LPS-stimulated macrophages are exposed to a ligand of a GPCR upstream to the cAMP pathway prematurely diverts macrophages to become anti-inflammatory and thus to diminish the innate immune response early at its inception. We recently evaluated the physiological effect of cAMP induction on IL-10 expression in a mouse septic shock model. We demonstrated that *in-vivo* administration of a macrophage-specific cAMP-elevating agent amplified early (but not late) LPS-induced IL-10 secretion to the serum, in accordance with the cell culture results (11). Selective knockout of β2-AR in innate immune cells in mice promotes death from sepsis in response to administration of an otherwise sub-lethal LPS dose, while co-administration of IL-10 rescues the mice (10). A rapid and synergistic induction of IL-10 serum levels was also demonstrated in a controlled human study, where subjects were administered LPS and epinephrine (65). These studies imply that the cAMP-inducing drug epinephrine, routinely used in the clinic for the treatment of sepsis (66), may have a protective effect in part via acceleration of IL-10 expression by macrophages in synergism with LPS. However, this treatment might exacerbate sepsis-induced immunoparalysis, a term describing an acquired anti-inflammatory state preventing the clearance of the primary infection and increasing the vulnerability to a secondary infection (66). Thus, timely and proportional IL-10 expression is critical to achieve a balance between inflammation and resolution.

## Data Availability

The datasets generated for this study are available on request to the corresponding author.

## Author Contributions

TZ conceived and coordinated the study and wrote the paper. OE and YG-G designed, performed, and analyzed most experiments, and OE also participated in writing the paper. BB and MA performed the experiment shown in Figure 1B. IB-D participated in several experiments. DG coordinated and YG designed, performed, and analyzed the experiments shown in Table 1. All authors reviewed the results and approved the final version of the manuscript.

### Conflict of Interest Statement

The authors declare that the research was conducted in the absence of any commercial or financial relationships that could be construed as a potential conflict of interest.

## Abbreviations

β-AR: β-adrenergic receptor
CRE: cAMP response element
interferon: IFN
Iso: isoproterenol
TF: transcription factor
TSS: transcription start site.
